# *Mycobacteriumabscessus*-associated vertebral osteomyelitis in an immunocompetent patient: a rare case report and literature review

**DOI:** 10.1038/s41394-019-0197-5

**Published:** 2019-05-31

**Authors:** Muhammad Z. Moral, Khusboo Desai, Abdul R. Arain, Robert E. O’Leary, Stefanos F. Haddad, James P. Lawrence

**Affiliations:** Division of Orthopedic Surgery, Albany Medical College, Albany, NY USA

**Keywords:** Infection, Bacterial infection

## Abstract

**Introduction:**

Vertebral osteomyelitis (VO) is an uncommon infection with Staphylococcus aureus as the most commonly implicated organism. VO caused by nontuberculous mycobacteria (NTM) such as *Mycobacterium* *abscessus* (*M*. *abscesscus)* is exceedingly rare with only eight cases reported in literature.

**Case presentation:**

We report a rare case of an 82-year-old male with a remote history of trauma who was diagnosed with NTM vertebral osteomyelitis. The patient initially underwent a vertebroplasty of T12 and kyphoplasty of L1 for pathologic compression fractures. Subsequent cultures revealed *M*. *abscess*us. The patient further underwent an anterior T12-L2 corpectomy and debridement with instrumented fusion, as well as a posterior T9-L4 instrumentation and fusion. He received multi-agent antibiotic therapy; however, was ultimately unable to tolerate the aggressive treatment regimen and his prolonged postoperative course.

**Discussion:**

Nontuberculous mycobacteria vertebral osteomyelitis is exceedingly rare. NTM vertebral osteomyelitis is challenging to treat. Surgical management plays a limited role in early VO, but is the mainstay treatment in chronic VO. Early recognition of the condition and shared patient management with multidisciplinary teams is key to successfully treating cases of NTM VO.

## Introduction

Since the first case was described in 1929 by Wilensky et al., vertebral osteomyelitis has remained relatively rare. A delay in diagnosis and treatment is associated with significant morbidity and mortality [1–3]. Although *S. aureus* is the most commonly isolated pathogen, other atypical organisms include Gram-negative *Bacilli, Streptococci*, as well as *Mycobacterium* species [4–8]. Treatment of vertebral osteomyelitis (VO) can be challenging, and includes both antibiotic treatment and surgical management [9]. Nontuberculous mycobacteria (NTM), particularly *Mycobacterium* *abscessus (M*. abscessus), is an extremely rare cause of vertebral osteomyelitis. *M*. *abscessus* is a rapidly growing mycobacterium found in soil, plants, and aqueous environments, including municipal drinking water and sewage systems [10–12]. It has been implicated as a cause of post-traumatic infections of the skin, soft tissue, and long bones. It has also been associated with chronic lung infections, endocarditis, keratitis, and disseminated disease in immunocompromised hosts [11]. To our knowledge, only eight cases of VO caused by M. *abscessus* have been reported in the literature, and four were in immunocompromised hosts [12, 13]. The treatment of NTM is difficult as it is not susceptible to most antituberculous drugs and, often requires a multi-agent drug therapy, including clarithromycin [10, 12]. We report a rare case of VO caused by *M. abscessus* in an immunocompetent individual residing in a non-mycobacterium endemic region.

## Case presentation

**Fig. 1:**
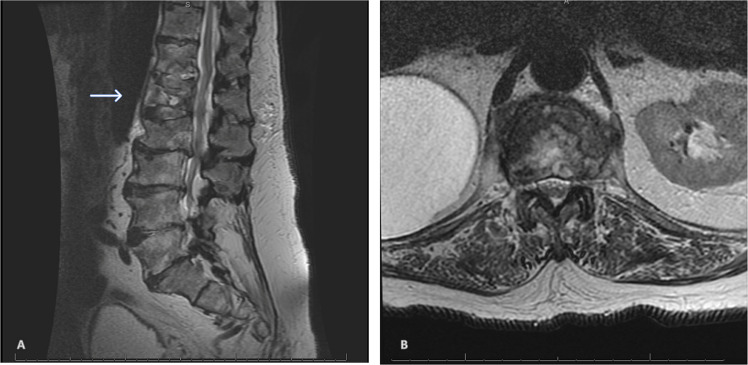
Sagittal (**a**) and axial (**b**) T2-weighted MRI with arrow pointing at an L1 vertebral body compression fracture concerning for infection or neoplasm. Markedly abnormal signal within the L1 vertebral body, with significant involvement of T12, and the superior aspect of L2

**Fig. 2:**
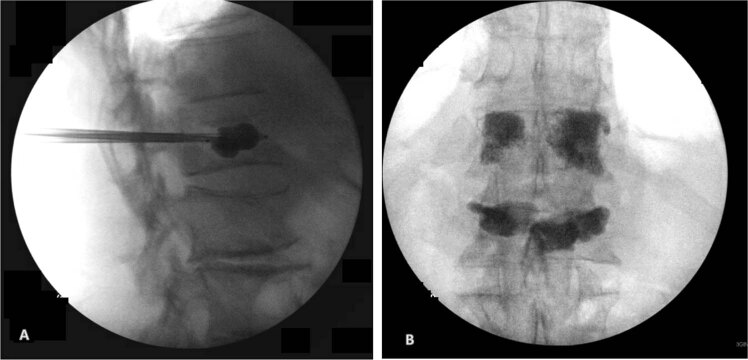
Intraoperative fluoroscopy of lateral (**a**) and anteroposterior (**b**) views of the thoracolumbar spine showing the injection of polymethylmethacrylate following the kyphoplasty of L1 and vertebroplasty of T12

An 82-year-old male presented with a remote history of prior falls and a recent traumatic compression fracture of his L1 vertebral body. He reported no previous fractures, weight loss or antecedent pain. He was initially treated with a brace and underwent physical therapy. At follow-up, he had no neurological deficits but demonstrated severe recurrence of pain with progressive functional impairment refractory to conservative management. An MRI showed a worsening L1 vertebral body collapse, a T12 inferior endplate fracture, and a L2 superior endplate fracture (Fig. 1). The imaging was suspicious for a pathologic etiology. With his intractable pain and collapse at the thoracolumbar junction, we proceeded with a kyphoplasty of L1, vertebroplasty of T12, and obtained a bone biopsy for pathology and culture (Fig. 2). The initial smear testing for acid-fast bacilli (AFB) was negative with an interim negative report at 2 weeks, and final identification of *M. abscessus* at 6 weeks. The infectious disease (ID) service was consulted for further management. The smear was presumed to be a contaminant given his history and presentation.

**Fig. 3:**
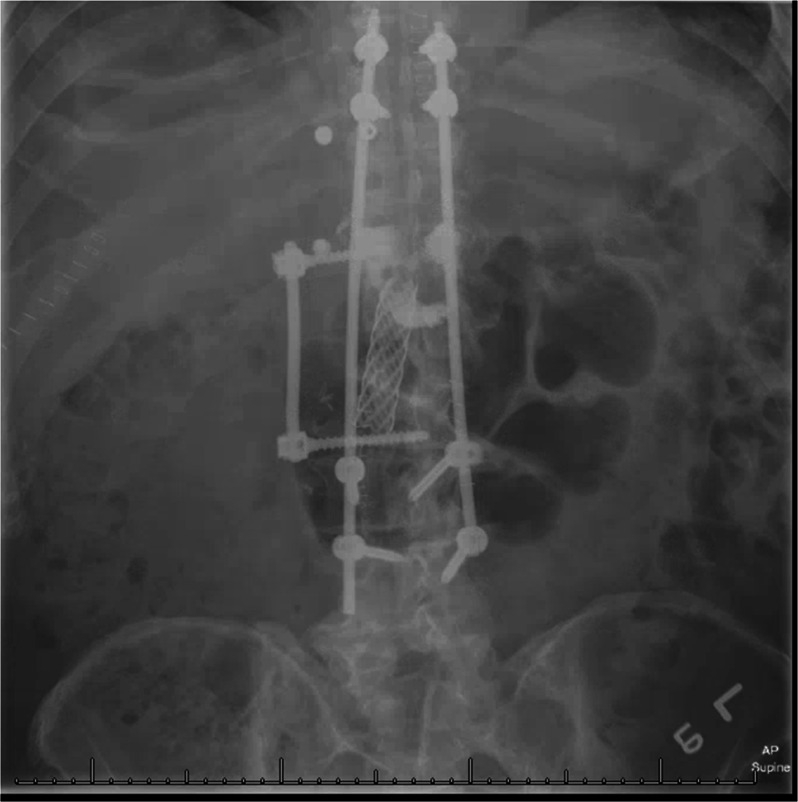
Upright anteroposterior radiograph of the thoracolumbar spine following an anterior corpectomy from T12-L2 and placement of an intervertebral biomechanical implant and stabilization with posterior T9-L4 instrumentation and fusion

At the 2-month follow-up, he remained neurologically intact, but continued to have functionally limiting pain. A repeat MRI demonstrated a psoas and paravertebral abscess with evidence of continued vertebral collapse. An image-guided bone biopsy confirmed the presence of *M. abscessus* and *Propionibacterium acnes*. Per the recommendations of the ID-team, he was started on Rocephin, then transitioned to a multi-agent regimen, including tigecycline, amikacin, meropenem, and oral-linezolid. After a completed course of antibiotics, he still continued to demonstrate clinical signs of infection with persistent pain. Again, advanced imaging confirmed progression of disease. He underwent an anterior T12-L2 corpectomy and debridement with instrumented fusion, as well as a posterior T9-L4 instrumentation and fusion (Fig. 3). Repeat AFB culture and smear redemonstrated *M. abscessus*. Postoperatively, he was placed on a regimen of amikacin, azithromycin, tigecycline, and meropenem.

His postoperative course was complicated by gastroparesis leading to poor oral intake and malnutrition requiring the total parental nutrition. In addition, he developed a right upper extremity deep venous thrombosis by his prior PICC line site. He required inpatient rehabilitation for functional retraining. The side effects of his multi-agent therapy included persistent nausea, antibiotic-induced *Clostridium difficile* colitis, and new-onset Barrett’s esophagus most likely secondary to tigecycline. Due to ongoing adverse treatment effects and poor quality of life, the patient and family elected to withdraw aggressive care and continue with palliative measures only.

## Discussion

The identification and diagnosis of VO presents many challenges to clinicians. It is an indolent and slowly progressing disease, often accompanied by nonspecific symptoms, such as back pain, malaise, and fatigue [14]. Although vertebral osteomyelitis is rare with an incidence of 2–4% of all cases of bone infections, it is a common public health concern in many developing countries. The most common microorganism associated with VO is *Staphylococcus aureus*. Other organisms implicated include *streptococci* species*, enterobacteriaceae, and enterococci*. In developing countries, tuberculosis is the most common cause of VO presenting with rapid destruction of vertebral bodies and early sparing of disc space [15].

A Pubmed review of the literature published between 1965 and 2017 demonstrated only eight cases of vertebral osteomyelitis caused by *M.* *abscessus*, half of which occurred in immunocompromised patients. Among the four immunocompromised patients, one received long-term steroid therapy for systemic lupus erythematous, while two had a history of intravenous (IV) drug abuse; one of which was diagnosed with Hepatitis C virus [11, 16, 17]. The final patient had undergone organ transplantation necessitating immunosuppressive therapy [18]. Among the four immunocompetent patients, two cases involved blunt trauma [15]. Only two cases in our literature search revealed an immunocompetent patient without any prior history of trauma or IV drug abuse; one of which resided in a TB-endemic region [19, 20].

Early diagnosis requires a thorough history, physical exam, laboratory testing, and imaging. Even in disseminated disease, blood cultures are often negative and offer limited value. Definitive diagnosis depends on bone and soft tissue sampling for culture and histologic examination [21]. Nontuberculous mycobacteria, including *M. abscessus*, are naturally occurring organisms found in water and soil and can inhabit body surfaces and secretions through the environment. In most immunocompetent individuals, exposure does not lead to disease. Therefore, isolated cultures of NTM can be often be considered colonizers or contaminants [20].

Recognition of the disease requires a high index of suspicion and an extensive workup. Prompt diagnosis ensures improved long-term outcomes, especially when the clinical course presents as an indolent, prolonged, and destructive process that is unresponsive to empirical antibacterial agents.

Our patient hailed from a non-endemic region and presented with nonspecific symptoms of back pain and a remote history of blunt trauma. Thus there was a low index of suspicion of osteomyelitis as the underlying cause. He continued to have pain while undergoing conservative treatment, prompting operative management and a bone biopsy. A second positive culture via image-guided bone biopsy redemonstrated *M. abscessus*, prompting initiation a multi-drug antibiotic regimen.

As in the majority of cases of vertebral osteomyelitis, initial treatment consists of medical and antibiotic therapy, in conjunction with external bracing. However, *M. abscessus* localized to the spine presents a unique challenge, as there is a lack of consensus on antimicrobial agents, combination therapies, and duration of treatment [22]. Case reports of *M. abscessus* are most commonly related to pulmonary or skin and soft tissue infections, with limited evidence of successful treatment in the spine. Moreover, *M. abscessus* has demonstrated resistance to standard antituberculous agents and antimicrobial therapies [10, 22].

The macrolide antibiotic clarithromycin is a mainstay of multi-agent therapy, it is often administered in combination with amikacin, cefoxitin, and tigecycline, but may also be delivered alone. Regimens often consist of weeks to months of parenteral treatment followed by oral therapy. However, certain isolates of *M. abscessus* are not susceptible to macrolide-based chemotherapy. Recent advances have been made possible by new molecular testing methods. Studies have determined that macrolide resistance is conferred by the *erm*(41) gene in some *M. abscessus* species, rendering macrolide-based chemotherapy against the organism futile. Thus molecular testing may further help guide treatment and predict outcomes [10, 22].

Surgery has a limited role in the early treatment of vertebral osteomyelitis. Indications are usually reserved for compression or instability leading to a neurologic deficit, or systemic sepsis with discernable foci [23]. However, the recommended mainstay of treatment for chronic vertebral osteomyelitis is an extensive surgical debridement and culture-directed antibiotic therapy.

At present, there are no standard guidelines for treating *M. abscessus* in the spine. While few prior cases have demonstrated successful outcomes with medical management alone [15, 17, 18, 24], other reports emphasize the necessity of adjunctive surgical management with appropriate antimicrobial therapy [11, 16, 19, 20]. Our study underscores the need for prompt identification and judicious antibiotic administration for *M. abscessus* vertebral osteomyelitis. However, appropriate patient selection when considering an extensive surgical procedure is also critical to achieving a satisfactory clinical outcome.
